# Increased Omega-3 Fatty Acid Intake Is Associated with Low Grip Strength in Elderly Korean Females

**DOI:** 10.3390/nu14122374

**Published:** 2022-06-08

**Authors:** Yun-Jung Bae, Xiang-Shun Cui, Seung-Ho Shin

**Affiliations:** 1 Major in Food and Nutrition, Division of Food Science and Biotechnology, Korea National University of Transportation, Jeungpyeong 27909, Korea; 2 Department of Animal Science, Chungbuk National University, Cheongju 28644, Korea; xscui@chungbuk.ac.kr; 3 Greengrassbio, Incorporated, Chungju 27462, Korea; somega3@naver.com

**Keywords:** eicosapentaenoic acid, docosahexaenoic acid, grip strength, sarcopenia, elderly

## Abstract

Omega-3 fatty acids, such as eicosapentaenoic acid (EPA) and docosahexaenoic acid (DHA), have anti-inflammatory properties and have recently been considered essential factors for maintaining muscle health. This study aimed to investigate the relationship between omega-3 fatty acid intakes and sarcopenia by assessing grip strength in elderly Koreans who are at risk of sarcopenia. This study was conducted on 5529 individuals (2449 males and 3080 females) aged ≥65 years from the raw data of the Korea National Health and Nutrition Examination Survey 2015–2019. In this study, we analyzed the association between EPA and DHA intake, calculated from a 24-h recall method data, and grip strength, a diagnostic criterion for sarcopenia. The cut-off values for low grip strength were <26 kg for males and <18 kg for females, which were set for the Asian population. The results indicated that elderly females consuming EPA and DHA below the adequate intake (AI) had significantly lower grip strength (*p* < 0.0001) and, had a higher percentage contribution from carbohydrates, but a significantly lower percentage contribution from protein (*p* < 0.0001), compared to elderly females consuming EPA and DHA at or above the AI. In addition, after adjusting for confounding factors, the odds of low grip strength were 0.777 times lower among elderly females consuming EPA and DHA at or above the AI than those consuming EPA and DHA below the AI (95% confidence interval: 0.616–0.979, *p* = 0.0322). These results suggest that sufficient intake of EPA and DHA is pivotal to mitigate a reduction in grip strength and to improve the quality of nutrient intake among elderly females.

## 1. Introduction

The elderly population has been increasing as a result of global demographic trends, and the number of people aged 60 years and older is expected to reach 2.1 billion, more than double its present size, by 2050 [1]. Therefore, it is important to maintain health and prevent morbidity in the elderly. In recent years, the interest in sarcopenia in the elderly has increased. Sarcopenia in older adults refers to a decrease in muscle mass as well as a decrease in muscle strength or physical performance as aging progresses [2]. Sarcopenia due to aging is closely related to frailty and may increase the risk of falls, fractures, metabolic diseases, and death [3,4].

Several factors have been reported to be associated with the development of sarcopenia, such as neuromuscular degeneration, changes in muscle protein turnover, changes in hormone levels and sensitivity, chronic inflammation, oxidative stress, and behavioral and lifestyle factors [5]. With regard to chronic inflammation and oxidative stress, it is known that chronic, low-grade inflammation tends to increase with age and both factors can activate inflammatory cascades in chronic or severe disease [6].

Accordingly, factors closely related to inflammation, omega-3 fatty acids, and sarcopenia have been widely investigated in recent years [7,8,9]. Long-chain omega-3 polyunsaturated fatty acids (omega-3 LCPUFAs) have been reported to prevent the loss of muscle mass and muscle strength, which are associated with aging, sarcopenia, and frailty [10]. In addition, omega-3 LCPUFAs modulate muscle protein synthesis, thereby enhancing muscle strength and physical performance [8,11].

Smith et al. [12] found that, in adults aged 60–85 years, 6 months of nutritional intervention with omega-3 fatty acids (1.86 g eicosapentaenoic acid (EPA) and 1.50 g docosahexaenoic acid (DHA)) extracted from fish oil significantly delayed the decrease in grip strength and thigh muscle volume. In addition, a similar study found that after 6 months of treatment with 1.50 g EPA and 1.50 g DHA, grip strength was unchanged, while the placebo group showed a significant decrease in grip strength [13]. On the other hand, Rolland et al. [14] reported that long-term intervention with omega-3 LCPUFAs in the elderly, according to a multidomain lifestyle intervention, did not have any significant effect on grip strength.

Findings from earlier studies on the relationship between sarcopenia and omega-3 fatty acid intake have been inconsistent due to various factors affecting sarcopenia, such as dietary and lifestyle factors. In previous studies conducted in Korea, the ratio of omega-3 LCPUFAs has been shown to be significantly higher among healthy elderly females than elderly females with sarcopenic obesity [15], and a high concentration of serum omega-3 LCPUFAs is positively correlated with muscle strength [16]. Nevertheless, there remains no in-depth analysis of the relationship between sarcopenia and omega-3 LCPUFAs in the elderly population in Korea.

Currently, the majority of Koreans elderly have a higher percentage of energy intake from carbohydrates but a lower percentage of energy intake from fat [17]. In particular, it has been reported that individuals aged 65 years and over have the lowest intake of omega-3 LCPUFAs among the adult population aged ≥ 19 years [18]. Given the low intake of omega-3 LCPUFAs in the elderly and the anti-inflammatory effects of omega-3 LCPUFAs, it is necessary to investigate the relationship between aging-induced sarcopenia and omega-3 fatty acid intake. This study aimed to analyze the relationship between omega-3 fatty acid intake and sarcopenia using grip strength in Korean individuals over 65 years of age.

## 2. Material and Methods

### 2.1. Data Source and Study Population

This study used raw data from the Korea National Health and Nutrition Examination Survey (KNHANES) 2015–2019. The overall weighted sample included 7250 adults aged ≥ 65 years. Subjects without grip strength data (*n* = 470) were excluded as well as subjects who reported implausible energy intake (<800 or >4000 kcal/d for males and <500 or >3500 kcal/d for females; *n* = 228) [19] and subjects with diseases such as cancer and rheumatoid arthritis (*n* = 1023), yielding a final sample size of 5529 (2449 males and 3080 females). The KNHANES is conducted by the government for public welfare and is thus deemed exempt from further review by the institutional review board under Subparagraph 1 of Article 2 of the Bioethics and Safety Act and under Article 2 (2) 1 of the Enforcement Rule of the same Act. The KNHANES was performed without IRB review between 2015 and 2017; however, from the 2018 KNHANES, in consideration of the collection of human biospecimens and release of raw datasets to third parties, IRB approval was obtained after review (2018-01-03-P-A, 2018-01-03-C-A), and all participants provided written informed consent prior to the study.

### 2.2. Data Collection

#### 2.2.1. General Characteristics

Age, sex, and general information (household income, education level, marital status, smoking status, drinking status, and physical activity) were acquired from health interview data. Annual household income was classified into low, mid-low, mid-high, and high income groups. Education level was classified as elementary school or lower, middle school, high school, and college or higher. Marital status was categorized as married or other (i.e., single, widowed, or separated). Smoking status was classified as current smoking (smoking ≥ 5 packs of cigarettes in their lifetime and currently smoking) or non-smoking. Drinking status was divided into drinking (drinking more than once per month in the past year) and non-drinking. If subjects reported walking for at least 30 min in a session for at least 5 days in the past week, they were considered to practice walking activity. If subjects reported muscle exercise on more than 2 days in the past week, they were considered to have practiced muscle exercise activity. Body mass index (BMI) was collected from health examination data. Subjects were divided into three categories using their BMI; normal (<23 kg/m^2^), overweight (≥23 and <25 kg/m^2^), and obese (≥25 kg/m^2^) [20].

#### 2.2.2. Grip Strength

Grip strength values were extracted from the examination data. Grip strength was measured using a digital dynamometer (Takei Digital Grip Strength Dynamometer, Model T.K.K. 5401, TAKEI, Tokyo, Japan), and subjects were asked to perform a grip strength test three times with each hand, alternating hands, starting with their dominant hand. In this study, the highest measured grip strength value was used [21].

#### 2.2.3. Assessment of Fat Intake

Nutrient intake was evaluated using nutrition survey data, which used the 24-h recall method for one day. In the nutrition survey, trained staff conducted individual interviews using measurement aids such as two-dimensional food containers and food models, measuring cups and spoons, 30 cm rulers, thickness sticks, and tape measures. Additionally, this study calculated the daily energy intake and percentage of energy intake from carbohydrates, proteins, and fats. For the fat intake assessment, the intake of total daily fat, saturated fatty acid (SFA), monounsaturated fatty acid (MUFA), polyunsaturated fatty acid (PUFA), omega-3 fatty acid, omega-6 fatty acid, cholesterol, α-linolenic acid (ALA), and EPA + DHA was analyzed, and the intake ratios of omega-6 fatty acids and omega-3 fatty acids were calculated. Fatty acid intake was calculated using a database by Korean Rural Development Administration [22] and another database built by Korea Disease Control and Prevention Agency [17]. In addition, the percentage of energy intake from SFA, MUFA, PUFA, omega-3 fatty acids, and omega-6 fatty acids were determined.

### 2.3. Statistical Analysis

All statistical analyses were performed using SAS version 9.4 (SAS Institute Inc., Cary, NC, USA) and a complex sample analysis method that incorporated sampling weights, clustering variables (PSU), and stratifying variables (kstrata). In order to examine the relationship between grip strength and EPA + DHA intake, this study divided subjects by sex, and then classified male and female subjects into subgroups based on EPA + DHA intake; mean intake at or above the adequate intake (AI) for EPA + DHA and mean intake below the AI for EPA + DHA. The AI for EPA + DHA is 310 mg/d in males 65–74 years old, 280 mg/d in males aged ≥ 75 years of age, 150 mg/d in females 65–74 years old, and 140 mg/d in females aged ≥ 75 years of age [23]. For the analysis, the general characteristics of subjects between the groups are shown as either mean and standard error or frequency and percentage. Differences between the groups were analyzed using linear regression analysis or Rao-Scott chi-square tests. For this analysis, general characteristics, such as age, BMI, household income, education level, marital status, smoking status, drinking status, and physical activity status, were adjusted. Moreover, this study assessed omega-3 fatty acids intake between the groups divided by grip strength, in which the cut-off points of grip strength in Asians (<26 kg for males and <18 kg for females) was applied [23]. The relationship between EPA + DHA intake and grip strength was analyzed using logistic regression analysis after adjusting for confounding factors, and the results are presented as odds ratios (OR) and 95% confidence intervals (CI). In all analyses, the significance level was set at *p* < 0.05.

## 3. Results

### 3.1. General Characteristics

Table 1 presents the comparison of general characteristics between the groups according to the intake of EPA + DHA. The average age was 72.51 years for males and 72.86 years for females (data not shown). Grip strength was significantly higher in males and females with a mean intake at or above the AI for EPA + DHA (*p* = 0.0010, *p* < 0.0001). BMI, BMI distribution, current smoking, and drinking status did not indicate any significant differences between the groups according to the intake level of EPA + DHA. The proportion of subjects with a ‘high’ household income was significantly higher in males and females with a mean intake at or above the AI for EPA + DHA (*p* < 0.0001, respectively). However, the proportion of subjects with a college or higher education level was significantly higher in the group with a mean intake at or above the AI for EPA + DHA for both males and females (*p* < 0.0001, respectively). In subjects with a mean intake at or above the AI for EPA + DHA, males had a higher proportion of those who practiced walking activities (*p* = 0.0255), and females had a higher proportion of those who were married (*p* = 0.0038), practiced walking activities (*p* = 0.0026), and practiced muscle exercise activity (*p* = 0.0073) compared to their counterparts.

### 3.2. Energy Distribution and Fat Intakes

Table 2 describes the intake results of energy nutrients and fat according to the intake of EPA + DHA. In the group with a mean intake at or above the AI for EPA + DHA, both males and females showed a significantly higher energy intake (*p* < 0.0001, respectively), but a significantly lower percentage of energy intake from carbohydrates and lower omega-6/omega-3 PUFA intake ratio (*p* < 0.0001) than the group with a mean intake below the AI for EPA + DHA. Furthermore, in females, the intake of total fat, MUFA, PUFA, omega-3 fatty acids, omega-6 fatty acids, cholesterol, ALA, and EPA + DHA, and the percentage of energy intake from these fatty acids were significantly higher in the group of subjects consuming EPA + DHA at or above the AI than the group of subjects consuming EPA + DHA below the AI. Table 3 shows fatty acid intake according to grip strength. The intake of total fat, SFA, MUFA, PUFA, omega-3 fatty acids, omega-6 fatty acids, cholesterol, ALA, and EPA + DHA, and the percentage of energy intake from these fatty acids were not significantly different between the groups according to grip strength. Additionally, the relationship between grip strength and the intake of omega-3 PUFA by composition was investigated (Supplement Table S1). The results indicated a positive correlation between the intake of α-linolenic acid and total omega-3 PUFA and grip strength in male subjects, and a positive correlation between the intake of DHA and grip strength in female subjects.

### 3.3. Relationship between EPA + DHA Intake and Grip Strength

Table 4 shows the results of the relationship between EPA + DHA intake and grip strength by sex. Model 1 shows the values without adjustment for confounding factors, while Model 2 shows the values after adjustment for age, BMI, household income, education level, marital status, smoking status, physical activity status, and energy intake. In males, there was no significant association between grip strength and EPA + DHA intake. However, after adjusting for confounding factors in females, the OR of low grip strength was 0.777 (95% CI, 0.616–0.979) in the group consuming EPA + DHA at or above the AI compared to the group consuming EPA + DHA below the AI (*p* = 0.0322).

## 4. Discussion

This study examined the relationship between omega-3 fatty acid intake and sarcopenia based on grip strength in elderly Korean individuals using recent large-scale national data to identify dietary factors that may alleviate sarcopenia. Sarcopenia is closely related to frailty, metabolic disease, and mortality in the elderly. The results showed that elderly females consuming EPA and DHA below the AI had significantly lower grip strength, a higher percentage of energy intake from carbohydrate, and a significantly lower percentage contribution of protein to total energy intake than those consuming EPA and DHA at or above the AI. In addition, after adjustment for confounding factors, the risk of low grip strength was significantly higher in elderly females consuming EPA and DHA below the AI than in their counterparts consuming EPA and DHA above the AI. From these results, we suggest that sufficient intake of EPA and DHA is important for mitigating decreases in grip strength and improving the quality of nutrient intake among elderly females.

Sarcopenia refers to a syndrome of decreased muscle mass, strength, and function due to aging; thus, various muscle indicators have been used for sarcopenia diagnosis [2,23,24]. The European Working Group on Sarcopenia in Older People (EWGSOP) developed a clinical definition and diagnostic criteria for sarcopenia across ages, combining appendicular skeletal muscle mass (ASM), muscle strength, and physical performance [24], with muscle strength measured as grip strength [25,26]. According to a study by Bhasin et al. [27], weakness, defined as low grip strength, and slowness, defined as a low usual gait speed, should be included in the definition of sarcopenia, and another study reported that grip strength is a very effective screening tool for sarcopenia [28]. Additionally, according to the Asian Working Group for Sarcopenia (AWGS) 2019 algorithm for sarcopenia, muscle strength, measured by grip strength, or physical performance, measured by 5-times chair stand test, determines possible sarcopenia, and severe sarcopenia is diagnosed by additional analysis of ASM [25]. This study investigated grip strength in 5529 elderly participants from a national epidemiological survey and determined that the proportion of subjects with low grip strength was 13.32% in elderly males and 30.83% in elderly females (data not shown). Based on these results, this study presents the current status of muscle health in elderly individuals at risk of age-related muscle loss.

Chronic low-grade inflammation causes many age-related changes [29,30]. Accordingly, recent studies have focused on the association between sarcopenia and LCPUFAs, a nutrient that helps reduce inflammation [8]. In their study on community-dwelling elderly, Robinson et al. [31] reported that the intake of fatty fish rich in omega-3 fatty acids had a significant positive effect on grip strength, while white fish intake was not related to grip strength. Some studies have found that inflammation can lead to severe muscle wasting in several disease states [32]. It is also known that reactive oxygen species and other inflammatory mediators can induce pro-inflammatory cytokines through NF-kB, and reactive oxygen species generated during oxidative stress can directly mediate muscle damage [33]. Therefore, omega-3 fatty acids are expected to help mitigate sarcopenia as an anti-inflammatory dietary factor. In addition, EPA and DHA, as omega-3 fatty acids, have been reported to exert positive effects on muscle fibers by their incorporation into membrane phospholipids of the sarcolemma and intracellular organs [34]. It has also been noted that a higher content of EPA and DHA in membrane phospholipids reduces the expression of factors that reduce the rate of muscle protein synthesis and regulate muscle protein breakdown [11]. These previous findings aid in the explanation for the significantly lower risk of low grip strength in elderly females consuming EPA and DHA at or above the AI in this study. Meanwhile, there was no relationship between EPA and DHA consumption and low grip strength in elderly males, probably due to the different dietary patterns of males and females. In this study, the proportion of elderly males consuming EPA and DHA below the AI was lower than that of elderly females, also, this difference may have been influenced by the general tendency of females to intake more anti-inflammatory dietary factors, such as fruits and vegetables, than males. There was a finding of different patterns of EPA and DHA supplementation between the sexes. When adults aged 20–70 were supplemented with moderate EPA and DHA intakes, there was a significantly increase in the concentration of EPA in blood among females than males [35]. Further, Da Boit et al. [36] reported that the positive effects of long-chain omega-3 fatty acids supplementation with resistance exercise occurred only in females and that sex differences were due to the differences in the enrichment of long-chain omega-3 fatty acids into cell membranes. Although the exact mechanism for the association is not fully elucidated here, as this study did not assess long-chain omega-3 fatty acids in the body, it is important for future studies to thoroughly investigate the relationship between long-chain omega-3 fatty acids content in the body and muscle health.

More recent evidence has revealed that muscle mass and malnutrition in the elderly have a close relationship [37]. Therefore, it is expected that a balanced intake of nutrients in the elderly can help preserve muscle strength and mass. In this study, among elderly females consuming EPA and DHA below the AI, the mean energy intake was 93.87% of the estimated energy requirements (EER), and among this group, the percentage of subjects with an energy intake below the EER was 70.60%. This implies an overall lower nutrient intake compared to elderly females consuming EPA and DHA at or above the AI (data not shown). Weight loss caused by insufficient energy intake can lead to a reduction in muscle mass by catabolism as well as depletion of stored fat reserves [38], acting as a negative factor in muscle health. Moreover, elderly females with an EPA and DHA intake below the AI had a higher percentage of energy intake from carbohydrates, but a lower percentage of energy intake from protein in this study. Dietary protein intake provides not only amino acids but also anabolic stimuli that are directly associated with muscle protein synthesis. Owing to the reduction of these metabolic reactions in the elderly, protein intake becomes relatively more important to maintain nitrogen balance as well as to prevent muscle mass and strength reductions. In the Dietary Reference Intakes for Koreans, in light of the risk of sarcopenia from low protein intake in the elderly, reference values for protein intake for those aged 75 years and older are the same as those aged 65–74 years [39]. Furthermore, EPA and DHA are abundant in fish, such as mackerel and saury, and these source foods are also rich in protein [39]. Therefore, it is thought that adequate intake of EPA and DHA foods in elderly females will contribute to protein intake and help mitigate sarcopenia.

This study had several limitations. First, given the cross-sectional design of this study, causality could not be concluded. Second, in this study, dietary data for the analysis of DHA and EPA intake were collected using a single 24-h recall, which is unable to account for daily variations to estimate usual dietary intake. However, in the nutrition survey in the KNHANES, individual recipes used to prepare foods were assessed and analyzed to calculate nutrient values, with the limited use of the existing food composition database, as a result, improving reliability of nutrition survey data [40,41]. Third, this study used only grip strength among the various sarcopenia indicators for the analysis. There are various sarcopenia indicators, such as muscle mass, walking speed, and grip strength. However, since only grip strength was measured in the KNHANES, other clinical sarcopenia indicators (e.g., walking speed) were not investigated due to the unavailability in this study. Nevertheless, being reported as an effective screening tool for sarcopenia [29], the measurement of grip strength is considered more important than the evaluation of muscle mass to predict clinical outcomes in the elderly [24,42]. In addition, as the data were from a national-scale survey with representative samples, the reliability of the data used is considered to be very high. Fourth, this study evaluated the association between grip strength and EPA and DHA based on EPA and DHA intake data alone. A previous study found that a high level of serum omega-3 fatty acids in the elderly was positively correlated with muscle strength [16], but the analysis of blood indicators was not performed in this study (DHA and EPA in blood was not analyzed in the KNHANES). Therefore, a detailed study on the relationship between DHA and EPA in blood and sarcopenia in a large population is needed. Fifth, people consuming a diet rich in EPA and DHA are more likely to have a healthy diet with a high content of bioactive components with anti-inflammatory actions, such as flavonoids [43,44]. However, this was not considered in this analysis. Despite these limitations, this study has high reliability using nationally representative data, and is the first study on the relationship between EPA and DHA intake and sarcopenia in elderly Korea.

In conclusion, our results showed that elderly females consuming EPA and DHA below the AI showed a higher percentage contribution of carbohydrate, but a significantly lower percentage contribution of protein to total energy intake, and a significantly higher risk of lower grip strength after adjustment for confounding factors, compared to elderly females consuming EPA and DHA at or above the AI. These results suggest that adequate intake of EPA and DHA can help mitigate the decrease in grip strength seen in the elderly. We recommend further well-designed, large-scale prospective studies on the relationship between sarcopenia and EPA and DHA intake.

## Figures and Tables

**Table 1:** General characteristics of the study subjects according to EPA and DHA intake.

		Male (*n* = 2449)			Female (*n* = 3080)		
		<AI (*n* = 1716)	≥AI (*n* = 733)	*p* Value	<AI (*n* = 2079)	≥AI (*n* = 1001)	*p* Value
Age (years)		72.67 ± 0.14	72.17 ± 0.22	0.0578	73.26 ± 0.14	72.04 ± 0.19	<0.0001
Body mass index (kg/m^2^)		23.72 ± 0.08	23.99 ± 0.12	0.0545	24.43 ± 0.08	24.20 ± 0.12	0.1026
Grip strength (kg)		33.13 ± 0.21	34.37 ± 0.30	0.0010	19.89 ± 0.14	20.98 ± 0.17	<0.0001
BMI distribution	<23 kg/m^2^	701 (40.08)	257 (37.09)	0.3442	732 (35.13)	367 (37.57)	0.4066
	≥23 and <25 kg/m^2^	465 (28.20)	205 (27.91)		509 (24.66)	248 (25.00)	
	≥25 kg/m^2^	550 (31.73)	271 (34.99)		838 (40.21)	386 (37.43)	
Household income	Quartile 1 (Low)	733 (41.94)	229 (30.10)	<0.0001	1134 (52.55)	452 (42.77)	<0.0001
	Quartile 2	531 (30.15)	232 (31.63)		525 (24.81)	275 (28.11)	
	Quartile 3	271 (16.78)	154 (21.96)		258 (14.49)	153 (16.01)	
	Quartile 4 (High)	171 (11.13)	111 (16.31)		152 (8.15)	116 (13.11)	
Education level	≤Elementary school	699 (42.48)	207 (30.44)	<0.0001	1437 (73.77)	602 (60.46)	<0.0001
	Middle school	279 (17.36)	129 (19.24)		218 (11.22)	141 (14.77)	
	High school	384 (24.71)	201 (29.02)		180 (10.54)	141 (16.73)	
	≥College	235 (15.45)	140 (21.30)		69 (4.47)	63 (8.05)	
Marital status	Married	1489 (87.26)	650 (90.01)	0.0983	1016 (47.78)	545 (54.39)	0.0038
	Others	227 (12.74)	83 (9.99)		1063 (52.22)	456 (45.61)	
Current smoking	Yes	306 (18.00)	126 (18.05)	0.9816	42 (2.32)	23 (2.81)	0.4902
Drinking	Yes	987 (58.40)	435 (61.87)	0.1623	366 (18.70)	182 (19.17)	0.7907
Walking activity	Yes	646 (42.10)	305 (48.02)	0.0255	599 (31.93)	345 (38.55)	0.0026
Muscle exercise activity	Yes	439 (28.41)	208 (31.66)	0.1818	152 (8.49)	99 (12.31)	0.0073

Values are shown as the mean ± standard error or number of case (%).

**Table 2:** Energy distribution and fat intakes of the study subjects according to EPA and DHA intake.

	Male (*n* = 2449)			Female (*n* = 3080)		
	<AI (*n* = 1716)	≥AI (*n* = 733)	*p* Value	<AI (*n* = 2079)	≥AI (*n* = 1001)	*p* Value
Energy (kcal)	1934.95 ± 29.09	2106.65 ± 37.99	<0.0001	1467.21 ± 29.91	1694.34 ± 33.11	<0.0001
Energy distribution						
Carbohydrate (%)	69.47 ± 0.44	66.65 ± 0.48	<.0001	69.40 ± 0.75	65.40 ± 0.75	<0.0001
Protein (%)	13.86 ± 0.16	16.16 ± 0.20	<.0001	13.32 ± 0.25	15.45 ± 0.27	<0.0001
Fat (%)	16.67 ± 0.35	17.18 ± 0.38	0.2020	17.28 ± 0.58	19.14 ± 0.57	<0.0001
PUFA (%) ^1^	4.53 ± 0.11	5.29 ± 0.13	<0.0001	4.84 ± 0.21	5.71 ± 0.21	<0.0001
MUFA (%) ^1^	5.12 ± 0.14	5.25 ± 0.15	0.4280	5.22 ± 0.22	5.86 ± 0.21	<0.0001
SFA (%) ^1^	5.25 ± 0.13	4.87 ± 0.14	0.0047	5.42 ± 0.18	5.64 ± 0.18	0.0742
Omega-3 PUFA (%) ^1^	0.74 ± 0.03	1.30 ± 0.05	<0.0001	0.78 ± 0.06	1.26 ± 0.06	<0.0001
Omega-6 PUFA (%) ^1^	3.81 ± 0.10	3.99 ± 0.10	0.0857	4.07 ± 0.18	4.46 ± 0.18	0.0003
Total fat (g)	35.90 ± 1.06	39.07 ± 1.18	0.0049	29.17 ± 1.23	35.50 ± 1.33	<0.0001
Total PUFA (g) ^1^	9.76 ± 0.32	11.97 ± 0.38	<0.0001	8.07 ± 0.43	10.48 ± 0.42	<0.0001
Total MUFA (g) ^1^	11.15 ± 0.40	11.97 ± 0.42	0.0474	8.91 ± 0.43	10.92 ± 0.47	<0.0001
Total SFA (g) ^1^	11.18 ± 0.36	11.05 ± 0.39	0.7261	9.17 ± 0.37	10.49 ± 0.41	<0.0001
Total cholesterol (mg)	172.10 ± 6.11	252.68 ± 9.96	<0.0001	132.33 ± 8.04	222.13 ± 8.62	<0.0001
α-linolenic acid (mg)	1386.12 ± 65.18	1580.24 ± 80.88	0.0203	1203.79 ± 90.73	1567.50 ± 87.42	<0.0001
EPA+DHA (mg) ^1^	141.76 ± 33.61	1120.07 ± 74.88	<0.0001	20.55 ± 22.98	564.09 ± 27.73	<0.0001
Total omega-3 PUFA (g) ^1^	1.57 ± 0.08	2.87 ± 0.12	<0.0001	1.25 ± 0.10	2.23 ± 0.09	<0.0001
Total omega-6 PUFA (g) ^1^	8.22 ± 0.28	9.09 ± 0.32	0.0018	6.83 ± 0.37	8.26 ± 0.37	<0.0001
Ratio of omega-6/omega-3 PUFA	7.68 ± 0.30	4.13 ± 0.22	<0.0001	7.20 ± 0.28	4.18 ± 0.25	<.0001

Values are shown as the mean ± standard error; adjusted for age, BMI, household income, education level, marital status, smoking status, drinking status, and physical activity status (walking and muscle exercise). ^1^ PUFA, polyunsaturated fatty acid; MUFA, monounsaturated fatty acid; SFA, saturated fatty acid; EPA, eicosapentaenoic acid; DHA, docosahexaenoic acid.

**Table 3:** Fat intakes of the study subjects according to grip strength.

	Male (*n* = 2449)			Female (*n* = 3080)		
	Low Grip Strength (*n* = 340)	Normal Grip Strength (*n* = 2109)	*p* Value	Low Grip Strength (*n* = 957)	Normal Grip Strength (*n* = 2123)	*p* Value
Total fat (g)	35.26 ± 1.72	37.24 ± 0.97	0.1766	31.06 ± 1.47	32.27 ± 1.21	0.1891
Total PUFA (g) ^1^	10.05 ± 0.60	10.61 ± 0.30	0.2917	8.91 ± 0.52	9.21 ± 0.40	0.3376
Total MUFA (g) ^1^	10.96 ± 0.63	11.50 ± 0.35	0.3224	9.58 ± 0.51	9.88 ± 0.43	0.3457
Total SFA (g) ^1^	10.71 ± 0.52	11.19 ± 0.33	0.2677	9.55 ± 0.44	9.82 ± 0.36	0.3554
Total cholesterol (mg)	196.11 ± 14.08	201.14 ± 6.19	0.7143	163.32 ± 9.49	175.14 ± 8.12	0.0576
α-linolenic acid (mg)	1321.49 ± 119.54	1471.33 ± 58.72	0.1642	1363.79 ± 105.97	1368.11 ± 83.56	0.9573
EPA + DHA (mg) ^1^	462.81 ± 58.99	490.68 ± 51.08	0.5574	236.73 ± 28.93	272.15 ± 25.7	0.1166
Total omega-3 PUFA (g) ^1^	1.88 ± 0.15	2.04 ± 0.08	0.2052	1.65 ± 0.11	1.70 ± 0.09	0.6014
Total omega-6 PUFA (g) ^1^	8.17 ± 0.50	8.57 ± 0.26	0.3601	7.27 ± 0.45	7.52 ± 0.35	0.3258
Ratio of omega-6/omega-3 PUFA ^1^	6.61 ± 0.40	6.40 ± 0.28	0.6467	6.09 ± 0.28	5.78 ± 0.26	0.2481

Values are shown as the mean ± standard error; adjusted for age, BMI, household income, education level, marital status, smoking status, drinking status, and physical activity status (walking and muscle exercise). ^1^ PUFA, polyunsaturated fatty acid; MUFA, monounsaturated fatty acid; SFA, saturated fatty acid; EPA, eicosapentaenoic acid; DHA, docosahexaenoic acid.

**Table 4:** Adjusted odd radios and 95% confidence intervals of low grip strength risk by EPA and DHA intakes.

		Male				Female			
		Crude		Adjusted		Crude		Adjusted	
		OR (95% CI)	*p* Value	OR (95% CI)	*p* Value	OR (95% CI)	*p* Value	OR (95% CI)	*p* Value
EPA and DHA ^1^	<AI	1		1		1		1	
intake	≥AI	0.687 (0.511–0.923)	0.0129	1.291 (0.900–1.853)	0.1644	0.631 (0.520–0.765)	<0.0001	0.777 (0.616–0.979)	0.0322

Adjusted for age, BMI, household income, education level, marital status, smoking status, drinking status, physical activity status (walking and muscle exercise), and energy intake; OR, odds ratio; CI, confidence interval. ^1^ EPA, eicosapentaenoic acid; DHA, docosahexaenoic acid.

## Data Availability

The KNHANES data used in the manuscript can be found at the following link: https://knhanes.kdca.go.kr/knhanes/main.do (accessed on 2 February 2022).

## Supplementary Materials

The following supporting information can be downloaded at: https://www.mdpi.com/article/10.3390/nu14122374/s1, Table S1: Relation between omega-3 fatty acid intake and grip strength by sex.
