# Supplementary material for: Increased Omega-3 Fatty Acid Intake Is Associated with Low Grip Strength in Elderly Korean Females
Source: Nutrients. 2022 Jun 8;14(12):2374. doi: 10.3390/nu14122374 (PMC9231389; doi:10.3390/nu14122374)
Supplement: Supplementary file 1 [file nutrients-14-02374-s001.zip › nutrients-1686181-supplementary.pdf]

Table S1. Relation between omega-3 fatty acid intake and grip strength by sex.

	Grip strength	
	Male	Female
α-linolenic acid	0.0002634**	0.0000100
EPA	-0.0003371	0.0005556
DHA	-0.0000474	0.0003947*
EPA+DHA	-0.0000632	0.0002467
Total omega-3 PUFA	0.1818593**	0.0302201

Total data was additionally adjusted for age, BMI, household income, education level, marital status, smoking status, drinking status, and physical activity status (walking and muscle exercise)

$p<0.05$ *: $p<0.01$ **
